# Clinical significance of filamin A in patients with acromegaly and its association with somatostatin and dopamine receptor profiles

**DOI:** 10.1038/s41598-018-37692-3

**Published:** 2019-02-04

**Authors:** Maria Caroline Alves Coelho, Marina Lipkin Vasquez, Luiz Eduardo Wildemberg, Mari C. Vázquez-Borrego, Luciana Bitana, Aline Helen da Silva Camacho, Débora Silva, Liana Lumi Ogino, Nina Ventura, Rafael Sánchez-Sánchez, Leila Chimelli, Leandro Kasuki, Raul M. Luque, Mônica R. Gadelha

**Affiliations:** 1 Neuroendocrinology Research Center/Endocrinology Division, Medical School and Hospital Universitário Clementino Fraga Filho, Universidade Federal do Rio de Janeiro, Rio de Janeiro, Brazil; 2 Endocrine Division, Hospital Universitário Pedro Ernesto, Universidade Estadual do Rio de Janeiro, Rio de Janeiro, Brazil; 3 Endocrine Division, Instituto Estadual de Diabetes e Endocrinologia Luiz Capriglione, Rio de Janeiro, Brazil; 4 Neuropathology and Molecular Genetics Laboratory, Instituto Estadual do Cérebro Paulo Niemeyer, Rio de Janeiro, Brazil; 5 Neuroendocrinology Division, Instituto Estadual do Cérebro Paulo Niemeyer, Rio de Janeiro, Brazil; 6 Maimonides Institute of Biomedical Research of Cordoba (IMIBIC), Córdoba, Spain; 7 Department of Cell Biology, Physiology and Immunology, University of Córdoba, Córdoba, Spain; 8 Hospital Universitario Reina Sofía, Córdoba, Spain; 9 Pathology Division, Instituto Nacional do Câncer, Rio de janeiro, Brazil; 10 Radiology Division, Instituto Estadual do Cérebro Paulo Niemeyer, Rio de Janeiro, Brazil; 11 Pathology Service, Reina Sofia University Hospital, Córdoba, Spain; 12 Endocrine Division, Hospital Federal de Bonsucesso, Rio de Janeiro, Brazil; 13 CIBER de la Fisiopatología de la Obesidad y Nutrición (CIBERobn), Madrid, Spain

## Abstract

Filamin-A (FLNA) plays a crucial role in somatostatin receptor (sst) subtype-2 signaling in somatotropinomas. Our objective was to investigate the *in vivo* association between FLNA and sst2 expression, sst5 expression, dopamine receptor subtype-2 (D2) expression, somatostatin receptor ligand (SRL) responsiveness and tumor invasiveness in somatotropinomas. Quantitative real-time PCR was used to evaluate the absolute mRNA copy numbers of *FLNA/sst2/sst5*/*D2* in 96 somatotropinomas. FLNA, sst2 and sst5 protein expression levels were also evaluated using immunohistochemistry. The Knosp-Steiner criteria were used to evaluate tumor invasiveness. Median *FLNA*, *sst2*, *sst5 and D2* copy numbers were 4,244, 731, 156 and 3,989, respectively. Thirty-one of the 35 available tumors (89%) were immune positive for FLNA in the cytoplasm and membrane but not in the nucleus. *FLNA* and *sst5* expression were positively correlated at the mRNA and protein levels (p < 0.001 and p = 0.033, respectively). *FLNA* was positively correlated with *sst2* mRNA in patients who were responsive to SRL (p = 0.014, R = 0.659). No association was found between *FLNA* and tumor invasiveness. Our findings show that in somatotropinomas FLNA expression positively correlated with *in vivo* sst5 and D2 expression. Notably, FLNA was only correlated with sst2 in patients who were controlled with SRL. FLNA was not associated with tumor invasiveness.

## Introduction

Three drug classes are used for the treatment of patients with acromegaly to reduce hormone secretion: somatostatin receptor ligands (SRLs), dopamine agonists (DA) and antagonists of growth hormone (GH) receptor^1,2^. SRLs decrease cell proliferation and induce apoptosis in somatotropinomas^1^. The first-generation SRLs, namely, octreotide and lanreotide, act predominantly on somatostatin receptor subtype 2 (sst2); pasireotide, a next-generation SRL, exhibits higher affinity to somatostatin receptor subtype 5 (sst5). Cabergoline is the only DA recommended for the treatment of acromegaly, and it binds to dopamine receptor subtype 2 (D2). First-generation SRLs are the first-line treatment for most acromegalic patients, but the biochemical response rate varies from 19 to 60%^3^. The mechanisms of SRL resistance are not fully elucidated^4^. Low sst2 expression is associated with resistance to SRLs, but some tumors with high sst2 expression are resistant, which suggests that additional factors are involved in SRL resistance^4–6^.

Previous studies by the group of Giovana Mantovani have demonstrated the important role of the cytoskeleton protein filamin A (FLNA) in sst2 expression and signaling in somatotropinomas^7–9^. FLNA is encoded by a gene located in chromosomal region Xq28, and it is a cytoskeletal protein that organizes actin filaments into stress fibers and networks^10^. This process is important for conformational changes at the cell membrane, where it acts as a key mediator of cytoskeleton reorganization^11^. FLNA binds diverse transmembrane proteins, such as G-protein-coupled receptors (GPCRs), ion channels and integrins, and anchors these proteins to the actin cytoskeleton; moreover, FLNA acts as an interface for protein-protein interactions^10,12–14^.

Peverelli and *et al*.^7^ demonstrated an association between FLNA expression and response to pharmacological therapy in somatotropinomas and suggested that a reduction of FLNA expression was another mechanism of resistance to SRLs, at least *in vitro*. Their study indicated that changes in FLNA expression altered the sst2 signaling pathway in somatotropinomas^7^. The same group recently demonstrated that sst2 inhibited rat and human tumoral somatotrophs migration and invasion *in vitro* via a molecular mechanism that involved FLNA-dependent cofilin recruitment and phosphorylation^9^. FLNA is also crucial for D2 expression and signaling in prolactinomas^15^. However, these results were demonstrated in *in vitro* cell models; no *in vivo* studies have confirmed the results.

Previous studies demonstrated that FLNA was involved in the control of cell mobility and extracellular matrix degradation in some tumoral tissues^16,17^ and FLNA knockdown enhanced metalloproteinase activity, which stimulated invasion, cancer cell migration and metastasis formation^16,18^. However, FLNA levels and its clinical relevance in somatotropinoma samples/patients were not examined. Therefore, the aim of this study was to analyze FLNA expression levels and its association with sst2, sst5 and D2 expression in human somatotropinoma samples and to investigate the association of FLNA expression with SRL responsiveness and tumor invasiveness in patients with acromegaly.

## Results

### Patient/sample characteristics

Ninety-six acromegaly patients were included in the present study [46 females; median age at diagnosis: 43 years old (15–75)]. Data regarding the tumor size at diagnosis were available in 72 patients [61 macroadenomas (85%)]. Tumor invasiveness was evaluated in 33 tumors, and 14 (42%) tumors were invasive adenomas based on MRI findings. There was no significant difference in age between patients harboring invasive adenomas [41 years old (22–63)] and patients harboring non-invasive adenomas [47 years old (28–75)].

Median GH level was 18.8 ng/mL (1.1–120) at time of diagnosis, and median IGF-I level was 325% ULNR (101–734). Data of treatment with first-generation SRL prior to surgery were not available in 21 patients. Sixty-two patients were treatment-naïve, and 13 patients were treated prior to surgery (2 of these patients were also treated with cabergoline). Nine patients used cabergoline after surgery. Radiotherapy was not performed in any patient prior to surgery.

Among the 96 patients who were included, data regarding responses to first-generation SRLs were available in 40 of the 96 included patients, and 23 (57.5%) of these 40 patients were controlled. One patient was excluded from the analysis of *sst2* and *sst5* mRNA levels because qPCR data were not obtainable due to the poor quality of the samples.

Granulation patterns were evaluated in 40 patients. Seventeen patients exhibited sparsely granulated tumors, and 23 patients exhibited densely granulated or mixed tumors.

### FLNA, sst2, sst5 and D2 mRNA expression levels

qPCR analyses of somatotropinoma tissues revealed that mRNA copy numbers of *FLNA*, *sst2*, *sst5 and D2* in somatotropinomas ranged from 821 to 147,992 (median 4,244), 12 to 23,954 (median 731), 0 to 5,555 (median 156), and 43 to 40,245 (median 3,989), respectively (Supplemental file Table 1).

### FLNA, sst2 and sst5 protein expression

**Figure 1:**
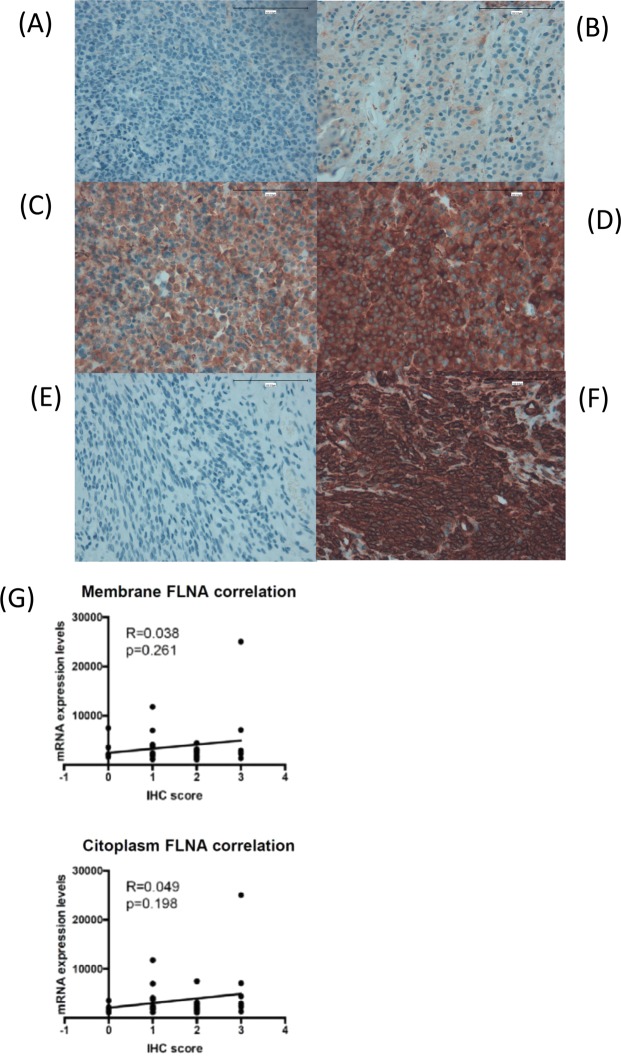
Immunohistochemical detection of filamin A (FLNA) in somatotropinomas. Representative images of FLNA immunohistochemical scores in somatotropinomas at 400X magnification: (**A**) Score-0: no membrane or cytoplasmic immunoreactivity; (**B**) Score 1: low membrane and cytoplasmic immunoreactivity; score 2: moderate membrane and cytoplasmic immunoreactivity; Score 3: high membrane and cytoplasmic immunoreactivity. Scale bar: 100 µm. In addition, negative (**E**) and positive (**F**) controls of the IHC validation are represented using a FFPE-uterus sample. (**G**) Correlation between FLNA IHC-staining and mRNA levels).

Immunohistochemistry (IHC) revealed that FLNA was expressed in 89% of the formalin-fixed paraffin-embedded (FFPE) somatotropinoma samples that were available for this analysis (n = 31 out of 35 samples). IHC also revealed that FLNA staining was low in 32% of the somatotropinomas (score-1; n = 10) compared with the moderate or intense staining found in 68% of the samples [score-2 (n = 12) and score-3 (n = 9)] (Fig. 1). Notably, FLNA was expressed at the membrane and cytoplasmic levels but not at the nuclear level in our cohort of somatotropinoma samples.

Sst2 and sst5 were expressed in 98% (39/40) and 95% (38/40) of patients, with median scores of 12(0–12) and 6(0–12), respectively. High sst2 and sst5 expression was found in 88% (n = 35) and 65% (n = 26) of patients, respectively (Supplemental file Table 1). No nuclear sst2 or sst5 immuno expression was observed.

### Correlation of FLNA with sst2, sst5 and D2 expression at the mRNA and/or protein levels

**Figure 2:**
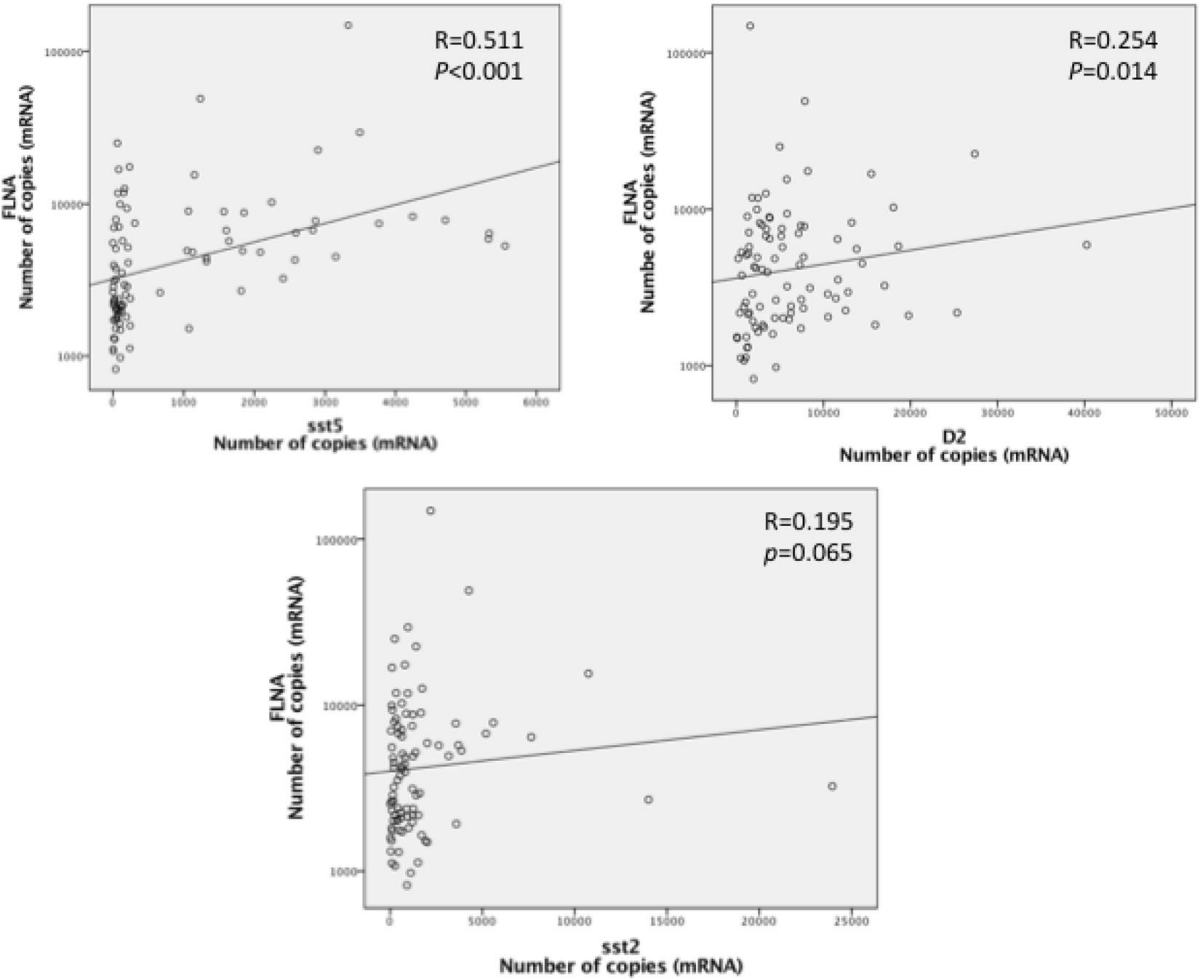
FLNA: filamin A, sst5: somatostatin receptor subtype 5; D2: dopamine receptor type 2; sst2: somatostatin receptor subtype 2. Graphics with logarithmic scale.

**Figure 3:**
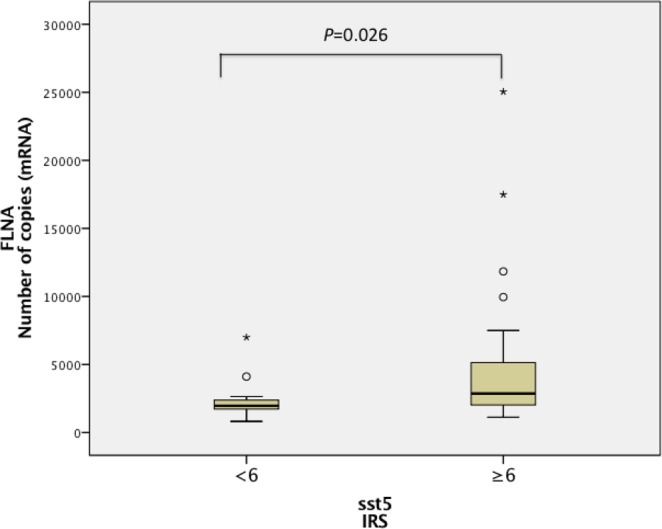
FLNA: Filamin A; sst5: somatostatin receptor subtype 5; IRS: immunoreactivity scoring system.

*FLNA* mRNA and protein levels were not correlated in this subset of patients (Fig. 1G). Notably, positive correlations between *FLNA* and *sst5* (p < 0.001, R = 0.511) and *D2* (p = 0.014, R = 0.254) mRNA expression were observed (Fig. 2). *FLNA* and *sst2* mRNA levels tended to be positively correlated, but this association did not reach statistical significance (p = 0.065, R = 0.195) (Fig. 2). *FLNA* mRNA levels were higher in patients with high sst5 IRS vs. patients with low sst5 IRS expression [2,869 (1,129–25,047) vs. 1,971 (820–6,998), p = 0.026] (Fig. 3). FLNA protein expression was associated with sst5 protein expression (p = 0.033). In contrast, no association between *FLNA* mRNA or protein expression and sst2 score was found in our cohort (p = 0.874 and p = 1.0, respectively).

S*st2* mRNA levels were higher in patients with high sst2 IRS compared to patients with low sst2 IRS expression [645(48−691) vs 79(12–99), p < 0.001], and *sst5* mRNA levels were also higher in patients with high sst5 IRS compared to patients with low expression [142(5–319) vs 36(0–214), p < 0.001] (Supplemental file Fig. 1).

### Correlations between FLNA mRNA or protein expression with patient/sample characteristics

No significant differences were observed in FLNA mRNA or protein levels between male and female patients or age at diagnosis. Similarly, no difference was found in FLNA mRNA or protein levels between invasive and non-invasive tumors. No association was observed between FLNA mRNA or protein levels and tumor size or granulation pattern.

### Effects of pre-surgical treatment with first-generation SRL on sst2, sst5, D2 and FLNA expression levels

*Sst2* mRNA expression was lower in patients treated with first-generation SRLs prior to surgery (n = 13) compared to patients who did not receive any pretreatment [96(12–23,954) × 937(34–13,995) respectively, p = 0.001]. The sst2 protein score was also lower in the pre-treated group [6(0–12) × 12^4–12^, respectively, p < 0.001]. However, pretreatment prior to surgery did not alter *FLNA*, *sst5* or *D2* mRNA levels and FLNA or sst5 protein levels.

### Association of FLNA with the pharmacological response to first-generation SRLs

There was no association between the response to first-generation SRLs and FLNA mRNA or protein levels. *Sst2* mRNA levels were higher in patients who achieved biochemical control vs. uncontrolled patients [1,204(92–23954) vs 231(12–13,995), p = 0.001].

**Figure 4:**
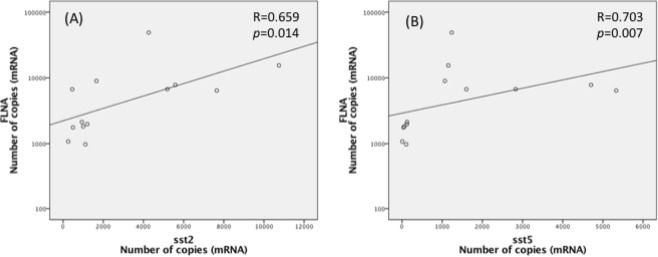
FLNA: Filamin A; sst5: somatostatin receptor subtype 5; sst2: somatostatin receptor subtype 2. Graphics with logarithmic scale.

**Figure 5:**
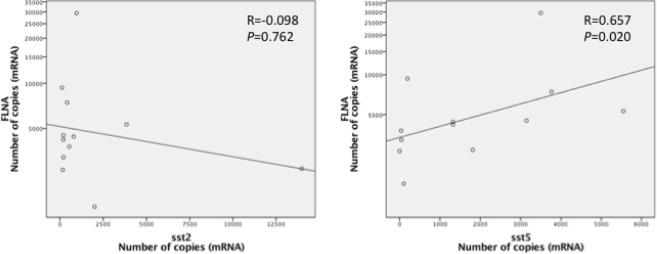
FLNA: filamin A; sst5: somatostatin receptor subtype 5; sst2: somatostatin receptor subtype 2. Graphics with logarithmic scale.

*Sst2* expression was lower in patients treated prior to surgery compared to patients who were not treated. Therefore, data from naïve and pretreated patients were analyzed separately. Positive correlations were observed between *FLNA* and *sst2* (p = 0.014, R = 0.659) and *sst5* (p = 0.007, R = 0.703) mRNA levels in controlled naïve patients (Fig. 4). In contrast, a positive correlation was only found between *FLNA* and *sst5* in uncontrolled patients (p = 0.020, R = 0.657) but not between FLNA and *sst2* (p = 0.762, R = −0.098) (Fig. 5).

No correlation between *FLNA* mRNA levels and *sst2* (p = 0.223, R = 0.363), *sst5* (p = 0.415, R = 0.247), or *D2* (p = 0.156, R = 0.418) mRNA expression was found in patients treated with SRLs prior to surgery.

## Discussion

Filamin A is a cytoskeletal protein that plays important roles in adhesion, conservation of cell shape, migration and intracellular signaling^10^. Our study evaluated the associations between the expression levels of FLNA (at the mRNA and protein level) and sst2, sst5 and D2 expression levels, the responsiveness to first-generation SRLs, and the presence of cavernous sinus invasion for the first time in patients with acromegaly.

No significant correlation between FLNA and sst2 expression was observed in the entire cohort, which is consistent with a previous report from Peverelli *et al*.^7^. However, we found a positive correlation between FLNA and sst2 in patients who were not pretreated and controlled with SRL therapy. Therefore, our current and previous data suggest that FLNA participates in the process of sst2 regulation and signaling in somatotropinomas. FLNA is involved in sst2 stabilization and signaling in tumoral somatotrophs, where it plays structural and functional roles^7,9^. The cyclic adenosine monophosphate (cAMP)/protein kinase A (PKA) pathway may be involved in this process and control FLNA stability via its phosphorylation status^19^. PKA phosphorylation of FLNA may produce conformational changes in regions involved in sst2 signaling and signal transduction pathways^19^. Therefore, one limitation of the present study is that we only assessed the association of FLNA expression with clinical features of the tumors independently of phosphorylation status.

FLNA scaffold function is necessary in somatotropinomas for sst2 to induce apoptosis and inhibit cell proliferation *in vitro*^7,9^. Peverelli *et al*.^7^ demonstrated that silencing of FLNA inhibited the ability of sst2 to activate caspase and reduce cyclin D1 levels in somatotropinoma primary cell cultures. FLNA stabilized sst2 expression via lysosomal degradation processes after prolonged agonist exposure *in vitro*^7^. Therefore, a correlation between FLNA and sst2 expression in patients treated with SRL prior to surgery was expected. However, the importance of this mechanism *in vivo* is not certain. Therefore, we analyzed expression levels of FLNA and sst2 in patients treated with SRLs prior to surgery, but no correlation between FLNA and sst2 was found. Notably, reduced expression levels of sst2 were found in patients treated with first-generation SRLs prior to surgery compared to patients who were not pre-treated, which is consistent with previous studies^20,21^. It is important to highlight a potential bias in our study because only patients who were not controlled with SRLs underwent surgery, and tumors with lower sst2 expression may have been selected for these analyses.

Notably, patient response to first-generation SRLs did not alter *FLNA* expression levels. However, *sst2* mRNA levels were higher in acromegaly patients who achieved biochemical response and disease control after SRL treatment, which is consistent with previous reports from our group^5,22^.

A partial association was found between FLNA and sst2 in our study, i.e., it was only observed in patients who were not pretreated and controlled with SRL. However, an association between *FLNA* and *sst5* levels was found regardless of SRL pretreatment or responsiveness. These results may be clinically relevant because sst5 is also involved in the inhibitory effects of somatostatin and SRL on GH release and cell proliferation^23^. The exact mechanism of sst5 activation and contribution to the response to first-generation SRL treatment of somatotropinomas is not certain. The data found in this study and previous studies of an association between FLNA and sst2^7,14,19^ suggest that FLNA is involved in the transcriptional and signaling regulation of sst5. However, further studies are required to elucidate the molecular mechanisms underlying this putative association. FLNA is required for the membrane localization of several G-protein coupled receptors via anchoring these proteins to the actin cytoskeleton^11^. FLNA gene silencing in parathyroid tumors reduced mRNA and protein expression of a calcium-sensing receptor^24^, which is a key signaling pathway involved in somatostatin and SRL actions via the activation of ssts, including sst5^25,26^. Higher sst5 expression is associated with a worse response to first-generation SRLs and a better response to pasireotide^22,24,27,28^. Gatto *et al*.^29^ demonstrated that a lower sst2/sst5 ratio was associated with a better response to pasireotide *in vitro* in GH secretion compared to octreotide. Further work is required to complete our understanding of this complex process and fully elucidate the molecular mechanisms underlying the putative association between FLNA and sst5 or sst2 in human somatotropinomas.

We found novel data of a positive correlation between *FLNA* and *D2* mRNA expression in somatotropinomas. Our results are partially consistent with a previous report that suggested an association of *FLNA* with D2 expression in a prolactinoma cell line (MMQ cells that expressed D2) but not in the somatotropinoma GH3 cell line (no D2 expression)^15^. This same study found that FLNA silencing in cultured prolactinoma cells decreased D2 protein expression and its migration to the plasma membrane, but it did not reduce its transcription^15^. FLNA also interacted with the N-terminal region of D2 (amino acids 211–241) *in vitro* in human melanoma cell lines, which increased the efficiency of D2 binding to the adenylate cyclase^30^. Li *et al*.^31^ demonstrated that FLNA was required for D2 cell surface localization in primary rat striatal cultures. Therefore, FLNA may command lysosomal degradation of D2 or its relocation to the membrane, and this process may be necessary for resensitization of desensitized D2. D2 silencing impaired extracellular signal-regulated kinase (ERK)1/2 phosphorylation and reduced prolactin release^15,32^. The decrease in FLNA expression in prolactinomas may be one mechanism that leads to DA resistance. We also demonstrated a positive correlation between FLNA and D2 expression in somatotropinomas. Therefore, we speculate that a decrease in FLNA may also be a mechanism of resistance to DA treatment in acromegaly. However, we could not evaluate this hypothesis in our study because only eleven patients were treated with cabergoline. Therefore, further studies with an ample cohort of patients may help clarify this relevant clinical question.

Finally, the involvement of FLNA in cancer progression via regulation of cell proliferation and migration was described previously in other tumors^33–35^. The absence of FLNA expression in prolactinomas impaired the inhibition of cell proliferation^15^. Despite the limited subset of cases included in our study, our data indicate that *FLNA* mRNA levels were not associated with the invasiveness features in our cohort of patients with somatotropinomas.

## Conclusion

Our data revealed that FLNA expression levels positively correlated with sst5 and D2, but not sst2, expression in somatotropinomas. However, a positive correlation between FLNA and sst2 was found in patients who were controlled with SRLs, which suggests that FLNA is important for sst2 signaling. We did not find any association of FLNA with tumor invasiveness. Therefore, the exact role of FLNA in somatotropinomas is not certain, and further studies are needed to better understand its connection to sst2, sst5 and D2 and its association with pharmacological treatment using drugs targeting these receptors, such as pasireotide and cabergoline.

### Subjects and methods

The Ethics Committee of Hospital Universitário Clementino Fraga Filho and Medical School/Universidade Federal do Rio de Janeiro approved this study. All participants and/or their legal guardians provided informed consent prior to entering the study. All methods and experimental protocols were performed in accordance with the approved guidelines and regulations of our institutions following the principles of the Declaration of Helsinki.

### Patients and tumors

Consecutive patients with acromegaly with available tumor samples after transsphenoidal surgery were included in the study. Patients underwent pituitary surgery between 2006 and 2015 in referral centers for pituitary disorders in Rio de Janeiro (Instituto Estadual do Cérebro Paulo Niemeyer and Hospital Universitário Clementino Fraga Filho). Tumor samples were immediately snap-frozen after surgery and stored at −80 °C for molecular analyses. Immunohistochemistry was performed in cases with available paraffin-embedding tumor samples.

Biochemical diagnosis of acromegaly was based on current criteria^36^. An expert neuroradiologist with experience in sellar MRI interpretation analyzed images. Tumor maximum diameter and the presence or absence of cavernous sinus invasion were analyzed according to modified Knosp–Steiner criteria^37^.

### Criteria for cure and response to somatostatin receptor ligands

Patients were considered not cured based on nadir GH levels higher than 1.0 ng/mL after an oral glucose tolerance test (OGTT) or with plasma IGF-I levels higher than age-matched normal levels three months after surgery. Biochemical response to medical treatment was assessed using GH and IGF-I levels after 6 months of treatment with octreotide LAR at a maximum dose of 30 mg or lanreotide autogel at a maximum dose of 120 mg. Patients with GH levels > 1.0 μg/L and/or IGF-I levels higher than age-matched normal levels were considered uncontrolled.

## Methods

### Hormonal assessment

Plasma GH levels were measured using a chemiluminescence assay kit (IMMULITE 2000; DPC - Diagnostic Products Corp., Inc., Los Angeles, CA, USA). The coefficients of variation (CV) inter- and intra-assay were 6.0 and 5.8%, respectively. The International Reference Preparation (IRP) for GH was 98/574.

Plasma IGF-I levels were measured using a chemiluminescence assay kit (IMMULITE 2000; DPC). The inter- and intra-assay CVs were 6.6 and 3.6%, respectively. The IRP for IGF-I was 87/518. IGF-I levels are expressed as a percentage of the upper limit of normal range (%ULNR).

### Immunohistochemistry

IHC staining was performed in FFPE samples using the Dako Envision system. The optimum antibody concentration for FLNA IHC analyses (1:150) using a commercially available human filamin antibody (Anti-Filamin A, Abcam, Cambridge, UK ab76289) was selected following antibody dilution tests (1:100; 1:150, 1:200) in uterus samples. A negative control image was taken from a uterus sample without the addition of primary antibody. IHC was performed in FFPE from somatotropinomas using standard procedures, as previously reported^38^.

Immunohistochemical analyses of sst2 and sst5 were performed using previously reported methods and reagents^39^. The CAM5.2 antibody (1:10000, Cell Marque, Rocklin, CA, USA, cat. number 452M-95) was used to evaluate the granulation patterns.

FLNA was scored based on staining intensity: 3, strong; 2, moderate; 1, mild; and 0, no staining. Scores of 2 and 3 were considered high expression. An immunoreactivity scoring system (IRS) was used to evaluate sst2 and sst5 immunoexpression, as previously reported^40^. Briefly, IRS was calculated as the product of percentage of positive cells (4, >80%; 3, 51–80%; 2, 10–50%; 1, <10%; 0, 0%) and intensity of staining (3, strong; 2, moderate; 1, mild; and 0, no staining). High IRS was defined as ≥6^40^. Tumors were classified as densely or sparsely granulated adenomas and “mixed tumors” as previously published^41^. Densely and mixed tumors were evaluated together because these tumors exhibit the same behavior. Two experts analyzed the samples, and a third researcher evaluated discordant results.

### Quantitative PCR

Total RNA was extracted from tumor samples using the Allprep® Universal kit (Qiagen, Hilden, Germany) according to the manufacturer’s protocol. Absolute mRNA copy number levels of *FLNA*, *sst2*, *sst5* and *D2* were analyzed using quantitative real-time RT-PCR (qPCR) and the Sybergreen® method, as previously described by our group^39^. Supplemental Table 2 lists the primers. The expression level (copy-number) of each of the transcripts analyzed was adjusted to a normalization factor of the expression levels of three housekeeping genes using the GeNorm 3.3 visual basic application to control for variations in the amount of RNA used and the efficiency of the reverse-transcription reaction^27^. The results are reported as gene copy number/NF.

### Statistical analysis

Statistical tests were performed using SPSS version 23.0 for Mac (IBM, Chicago, IL, USA). The results are reported as median values (minimum–maximum). The Mann–Whitney U non-parametric test was used to compare numeric variables between groups, and correlation coefficients were calculated using Spearman rank order R. Fisher’s exact test or the χ^2^ test was used to compare frequencies between groups according to sample size. A p value < 0.05 was considered significant.

## Supplementary information

Supplemental file
