# Supplementary material for: Clinical significance of filamin A in patients with acromegaly and its association with somatostatin and dopamine receptor profiles
Source: Sci Rep. 2019 Feb 4;9:1122. doi: 10.1038/s41598-018-37692-3 (PMC6361919; doi:10.1038/s41598-018-37692-3)
Supplement: Supplementary file 1 — Supplemental file [file 41598_2018_37692_MOESM1_ESM.docx]

**Title**

**Clinical significance of filamin A in patients with acromegaly and its association with somatostatin and dopamine receptor profiles**

**Authors:**

**Maria Caroline Alves Coelho, Marina Lipkin Vasquez, Luiz Eduardo Wildemberg, Mari C.Vázquez-Borrego, Luciana Bitana, Aline Helen da Silva Camacho, Débora Silva, Liana Lumi Ogino, Nina Ventura, Rafael Sánchez-Sánchez; Leila Chimelli, Leandro Kasuki, Raul M. Luque, Mônica R. Gadelha**

**Supplementary Table 1.** *FLNA*, *sst2*, *sst5*, *D2* mRNA levels in individual somatotropinomas estimated mRNA copy number corrected by a normalization factor (NF) derived from the expression of three housekeeping genes (glyceraldehyde-3-phosphate dehydrogenase - GAPDH, β-actin - ACTB and hypoxanthine-guanine phosphoribosyl transferase – HPRT) together with sst2 and sst5 protein evaluation (as IRS).The blanks represent data not available.

| No. | FLNA/NF | sst2/NF | sst5/NF | D2/NF | NF | sst2 IRS | sst5 IRS |
| --- | --- | --- | --- | --- | --- | --- | --- |
| 1 | 1754 | 113 | 60 | 2158 | 2.86 |  |  |
| 2 | 1976 | 1204 | 120 | 6048 | 1.15 | 12 | 6 |
| 3 | 12603 | 1731 | 164 | 3370 | 0.94 |  |  |
| 4 | 1076 | 255 | 6 | 855 | 2.79 |  |  |
| 5 | 5091 | 670 | 40 | 1112 | 0.78 | 12 | 9 |
| 6 | 2253 | 577 | 10 | 12529 | 2.99 | 12 | 1 |
| 7 | 976 | 1110 | 103 | 4527 | 2.39 |  |  |
| 8 | 3775 | 541 | 38 | 601 | 0.06 | 8 | 6 |
| 9 | 1129 | 1505 | 239 | 1064 | 1.08 | 12 | 12 |
| 10 | 1527 | 69 | 38 | 1123 | 4.12 | 6 | 4 |
| 11 | 2645 | 164 | 0 | 7438 | 1.53 | 9 | 0 |
| 12 | 7492 | 1194 | 309 | 5208 | 1.00 | 12 | 12 |
| 13 | 9358 | 120 | 197 | 5803 | 0.32 |  |  |
| 14 | 16824 | 97 | 81 | 15483 | 0.64 |  |  |
| 15 | 2542 | 12 | 178 | 1047 | 0.59 | 4 | 12 |
| 16 | 2016 | 380 | 40 | 5348 | 2.08 | 8 | 4 |
| 17 | 1732 | 645 | 8 | 7404 | 2.44 | 12 | 2 |
| 18 | 2046 | 404 | 65 | 10513 | 2.80 | 12 | 9 |
| 19 | 2950 | 1597 | 160 | 12808 | 2.76 | 12 | 6 |
| 20 | 25047 | 241 | 62 | 4959 | 0.81 | 6 | 6 |
| 21 | 820 | 906 | 39 | 1952 | 0.68 | 12 | 4 |
| 22 | 2625 | 63 | 666 | 4519 | 3.75 | 6 | 9 |
| 23 | 2176 | 236 | 142 | 1467 | 2.26 | 6 | 9 |
| 24 | 2017 | 166 | 91 | 4393 | 1.42 | 6 | 9 |
| 25 | 1761 | 492 | 34 | 3179 | 2.65 | 12 | 2 |
| 26 | 1495 | 2002 | 105 | 76 | 1.44 |  |  |
| 27 | 1646 | 1701 | 98 | 2472 | 1.05 | 12 | 9 |
| 28 | 1524 | 1901 | 1076 | 43 | 1.29 | 12 | 12 |
| 29 | 2131 | 937 | 123 | 1319 | 2.63 |  |  |
| 30 | 2374 | 892 | 83 | 824 | 2.15 |  |  |
| 31 | 2398 | 428 | 245 | 6261 | 0.66 |  |  |
| 32 | 1119 | 96 | 5 | 481 | 1.29 | 12 | 2 |
| 33 | 3534 | 401 | 125 | 11652 | 1.65 |  |  |
| 34 | 4411 | 792 | 1319 | 7254 | 0.03 | 12 | 12 |
| 35 | 1817 | 79 | 189 | 2997 | 4.08 | 4 | 12 |
| 36 | 2326 | 99 | 15 | 7682 | 2.44 | 4 | 6 |
| 37 | 5731 | 3691 | 136 | 1451 | 1.25 | 12 | 8 |
| 38 | 2380 | 1240 | 27 | 2680 | 2.35 | 12 | 4 |
| 39 | 2175 | 1527 | 98 | 369 | 1.48 | 12 | 6 |
| 40 | 2877 | 137 | 201 | 1847 | 0.74 | 12 | 12 |
| 41 | 1822 | 999 | 58 | 15933 | 2.35 | 9 | 2 |
| 42 | 1927 | 3578 | 80 | 1877 | 0.57 | 12 | 4 |
| 43 | 1593 | 17 | 244 | 4157 | 3.90 | 0 | 6 |
| 44 | 3210 | 199 | 46 | 5817 | 2.41 |  |  |
| 45 | 1318 | 34 | 11 | 1281 | 5.33 |  |  |
| 46 | 2094 | 597 | 45 | 19781 | 4.72 | 6 | 2 |
| 47 | 4113 | 637 | 214 | 2938 | 0.01 | 9 | 0 |
| 48 | 6998 | 48 | 14 | 7131 | 0.54 | 9 | 4 |
| 49 | 11780 | 950 | 70 | 2471 | 0.68 |  |  |
| 50 | 3134 | 1206 | 13 | 8432 | 1.78 | 9 | 6 |
| 51 | 5197 | 1371 | 210 | 1382 | 1.16 | 12 | 12 |
| 52 | 2177 | 1227 | 8 | 6236 | 5.23 |  |  |
| 53 | 11838 | 319 | 152 | 1782 | 1.35 | 6 | 6 |
| 54 | 2869 | 1406 | 5 | 10482 | 1.98 | 12 | 6 |
| 55 | 17487 | 795 | 234 | 8188 | 0.19 |  |  |
| 56 | 9954 | 86 | 100 | 2336 | 0.26 | 1 | 6 |
| 57 | 7090 | 637 | 84 | 1425 | 1.51 |  |  |
| 58 | 3958 | 817 | 11 | 3541 | 1.03 |  |  |
| 59 | 1303 | 462 | 27 | 1217 | 1.45 |  |  |
| 60 | 15489 | 10747 | 1152 | 5762 | 0.17 |  |  |
| 61 | 29408 | 961 | 3497 |  | 0.05 |  |  |
| 62 | 4287 | 637 | 2574 | 2017 | 0.47 |  |  |
| 63 | 5903 | 2006 | 5321 | 40245 | 0.77 |  |  |
| 64 | 6717 | 452 | 1601 | 5130 | 7.97 |  |  |
| 65 | 147992 | 2189 | 3330 | 1567 | 0.06 |  |  |
| 66 | 8160 |  |  | 2668 | 0.07 |  |  |
| 67 | 6738 | 5189 | 2826 | 3359 | 0.90 |  |  |
| 68 | 2182 |  |  | 25349 | 0.06 |  |  |
| 69 | 5560 | 92 | 0 | 13789 | 2.16 |  |  |
| 70 | 6890 |  |  |  | 0.03 |  |  |
| 71 | 5800 |  |  | 18604 | 0.29 |  |  |
| 72 | 22555 | 1401 | 2900 | 27383 | 0.21 |  |  |
| 73 | 7761 | 3546 | 2864 | 7791 | 0.09 |  |  |
| 74 | 2695 | 13995 | 1813 | 11413 | 7.46 |  |  |
| 75 | 4943 | 3163 | 1046 | 7703 | 8.12 |  |  |
| 76 | 6419 | 7648 | 5332 | 11604 | 0.33 |  |  |
| 77 | 48922 | 4260 | 1235 | 7849 | 0.06 |  |  |
| 78 | 8211 |  |  | 13234 | 4.83 |  |  |
| 79 | 7442 | 416 | 3767 | 3439 | 1.07 |  |  |
| 80 | 8772 | 1225 | 1852 | 3821 | 1.24 |  |  |
| 81 | 7911 | 220 | 41 | 2951 | 6.89 |  |  |
| 82 | 4202 | 194 | 1322 | 2210 | 6.19 |  |  |
| 83 | 4829 | 159 | 2085 | 4382 | 5.98 |  |  |
| 84 | 4424 |  |  |  | 1.39 |  |  |
| 85 | 8285 | 321 | 4242 |  | 1.47 |  |  |
| 86 | 8984 | 1662 | 1067 | 1238 | 0.26 |  |  |
| 87 | 4499 | 205 | 3150 | 14440 | 6.38 |  |  |
| 88 | 4918 | 1230 | 1833 | 2390 | 5.76 |  |  |
| 89 | 6471 | 641 | 2586 | 3807 | 6.60 |  |  |
| 90 | 10263 | 637 | 2246 | 18036 | 7.81 |  |  |
| 91 | 5714 | 2625 | 1639 | 5276 | 7.29 |  |  |
| 92 | 8903 | 864 | 1567 | 3759 | 5.19 |  |  |
| 93 | 4833 | 801 | 1124 | 190 | 5.76 |  |  |
| 94 | 3245 | 23954 | 2407 | 17016 | 3.12 |  |  |
| 95 | 5318 | 3851 | 5555 | 586 | 5.11 |  |  |
| 96 | 7839 | 5586 | 4704 | 7373 | 0.07 |  |  |
| Median | 4244 | 731 | 156 | 3989 |  | 12 | 6 |

FLNA: filamin A, sst: somatostatin receptor, D2: dopamine receptor, NF: normalization factor, IRS: immunoreactivity scoring system. Blank cells represent unavailable data.

**Supplementary Table 2.** Primer sequences, product sizes and GenBank Accession numbers used for quantitative assessment of housekeeping genes (GAPDH, β-actinand HPRT), sst2, sst5, D2 and FLNA by RT-qPCR.

| Gene | Sense | Anti-sense | Product size (pb) | Genbank Accession nº. |
| --- | --- | --- | --- | --- |
| ACTB | ACTCTTCCAGCCTTCCTTCCT | CAGTGATCTCCTTCTGCATCCT | 176 | NM_001101 |
| HPRT1 | CTGAGGATTTGGAAAGGGTGT | TAATCCAGCAGGTCAGCAAAG | 157 | BT019350 |
| GAPDH | AATCCCATCACCATCTTCCA | AAATGAGCCCCAGCCTTC | 122 | NM_002046 |
| Sst2 | GGCATGTTTGACTTTGTGGTG | GTCTCATTCAGCCGGGATTT | 185 | NM_001050 |
| Sst5 | CTGGTGTTTGCGGGATGTT | GAAGCTCTGGCGGAAGTTGT | 183 | NM_001053 |
| D2 | CGAGCATCCTGAACTTGTGTG | GCGTTATTGAGTCCGAAGAGG | 172 | NM_016574 |
| FLNA | CCATGACAACACCTACACAGTCA | CTTGGAGATACTGCCACTGAGAA | 120 | NM_001110556 |

ACTB: β-actin, HPRT: hypoxanthine-guaninephosphoribosyl transferase, GAPDH: glyceraldehyde-3-phosphate dehydrogenase, Sst: somatostatin receptor, D2: dopamine receptor type 2, FLNA: filamin A.

**Supplementary Figure 1**: Comparision of *sst2* and *sst5*mRNA expression with protein expression


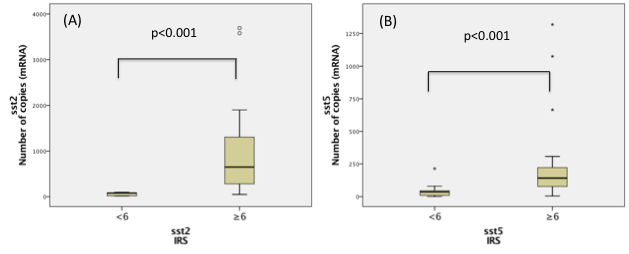


(A) *sst2* mRNA expression in patients with low sst2 (<6) IRS versus high IRS (≥6) (B) *sst5* mRNA expression in patients with low sst5 IRS (<6) versus high IRS (≥6).sst: somatostatin receptor, IRS: immunoreactivity scoring system
